# Vaccine effectiveness against infection with the Delta (B.1.617.2) variant, Norway, April to August 2021

**DOI:** 10.2807/1560-7917.ES.2021.26.35.2100793

**Published:** 2021-09-02

**Authors:** Elina Seppälä, Lamprini Veneti, Jostein Starrfelt, Anders Skyrud Danielsen, Karoline Bragstad, Olav Hungnes, Arne Michael Taxt, Sara Viksmoen Watle, Hinta Meijerink

**Affiliations:** 1 Department of infectious disease control and vaccines, Norwegian Institute of Public Health, Oslo, Norway; 2 Department of infectious disease control and preparedness, Norwegian Institute of Public Health, Oslo, Norway; 3 Department of virology, Norwegian Institute of Public Health, Oslo, Norway; 4 ECDC Fellowship Programme, Field Epidemiology path (EPIET), European Centre for Disease Prevention and Control, (ECDC), Stockholm, Sweden

**Keywords:** *:* COVID-19 Vaccines, SARS-CoV-2, Norway, Delta variant of concern, Vaccine effectiveness, B.1.617.2

## Abstract

Some variants of SARS-CoV-2 are associated with increased transmissibility, increased disease severity or decreased vaccine effectiveness (VE). In this population-based cohort study (n = 4,204,859), the Delta variant was identified in 5,430 (0.13%) individuals, of whom 84 were admitted to hospital. VE against laboratory confirmed infection with the Delta variant was 22.4% among partly vaccinated (95% confidence interval (CI): 17.0−27.4) and 64.6% (95% CI: 60.6−68.2) among fully vaccinated individuals, compared with 54.5% (95% CI: 50.4−58.3) and 84.4% (95%CI: 81.8−86.5) against the Alpha variant.

Since 2020, several variants of concern of severe acute respiratory syndrome coronavirus 2 (SARS-CoV-2) that are associated with increased transmissibility, disease severity or potentially decreased vaccine effectiveness (VE) have been identified [1]. First detected in India in December 2020, the Delta variant (Phylogenetic Assignment of Named Global Outbreak (Pango) lineage designation B.1.617.2) was reported by 124 countries by the end of July 2021 and is expected to outcompete other currently circulating variants [2,3].

Preliminary reports suggest decreased VE against infection with the Delta variant compared with the Alpha variant, while VE against severe disease seems to be maintained [4,5]. In order to understand the impact of the increased circulation of the Delta variant, and to inform policy on vaccination and non-pharmaceutical interventions in Norway, we estimated the effectiveness of coronavirus disease (COVID-19) vaccines against infection with the Delta variant compared with the Alpha variant in a population-based cohort study.

## Retrieving data

In Norway, the Delta variant was first identified in mid-April (week 15) 2021 and accounted for 67% of all sequenced samples by mid-July (week 28), overtaking the Alpha variant (B.1.1.7) as the dominating variant [6].

We obtained data on 19 August 2021 from BeredtC19, a national emergency preparedness registry established to monitor SARS-CoV-2 infection and the use of health services in Norway. The registry contains individual-level data from various Norwegian registries (Supplement, Table S1) [7]. We included all adults aged 18 years or older with a national identification number registered in Norway. We obtained data from 15 April to 15 August 2021 that were right-censored at a positive SARS-CoV-2 test (with or without sequencing), death or end of follow-up (15 August 2021) for this study [8]. We defined the outcome as infection with Delta or Alpha variant confirmed by PCR or by whole genome sequencing. The laboratory testing process for variants of SARS-CoV-2 in Norway has been described in detail elsewhere [6,9]. We excluded individuals with prior SARS-CoV-2 infection, and those with an interval between first and second dose of a COVID-19 vaccine (Table 1) that did not adhere to national recommendations. The official recommendation for time between doses in Norway is 12 weeks (minimum 21 days) for Comirnaty (BioNTech-Pfizer, Mainz, Germany/New York, United States) and 12 weeks for Spikevax (minimum 28 days) (mRNA-1273, Moderna, Cambridge, United States) for immunocompetent individuals, while for immunocompromised individuals the recommended interval is 3 and 4 weeks, respectively [10]. Underlying conditions were categorised as ‘high risk’ or ‘medium risk’ as stipulated by the national vaccination programme (Supplement, part 1) [8]. Vaccination status was defined as: unvaccinated (unvaccinated and/or < 21 days after first vaccine dose), partly vaccinated ( ≥ 21 days after first vaccine dose and/or  < 7 days after second vaccine dose and fully vaccinated ( ≥ 7 days after second vaccine dose).

**Table 1:** Characteristics of the study population and of those who tested positive for SARS-CoV-2 Alpha or Delta variant, Norway, 15 April−15 August 2021 (n = 4,204,859)

Characteristics	Study population (n = 4,204,859)		Variant of SARS-CoV-2			
			Alpha (n = 13,001)		Delta (n = 5,430)	
	%	n	%	n	%	n
**Sex**						
Female	49.9	2,096,298	46.7	6,076	44.9	2,436
Male	50.1	2,108,561	53.3	6,925	55.1	2,994
**Age groups in years**						
18−24	10.7	448,515	32.4	4,215	29.1	1,579
25−34	17.4	729,462	22.1	2,871	32.3	1,753
35−44	16.4	689,615	18.1	2,357	18.0	979
45−54	17.4	729,622	16.6	2,152	12.7	688
55−64	15.3	642,009	7.4	956	4.6	250
65−74	12.7	534,921	2.4	309	1.8	98
75−84	7.5	313,779	0.6	83	1.0	55
≥85	2.8	116,936	0.5	58	0.5	28
**Country of birth**						
Norway	74.6	3,137,823	64.5	8,386	61.5	3,339
Outside of Norway	25.4	1,066,410	35.5	4,611	38.4	2,086
Unknown	0.01	626	0.03	4	0.09	9
**County of residence**						
Oslo	12.8	538,714	25.3	3,291	21.0	1,141
Rogaland	8.7	366,725	9.6	1,242	4.2	229
Møre and Romsdal	5.0	208,663	1.5	193	3.8	207
Nordland	4.6	192,682	1.0	131	1.3	70
Viken	22.9	960,836	20.3	2,639	24.6	1,334
Innlandet	7.1	298,949	5.4	703	3.7	202
Vestfold and Telemark	8.0	334,750	12.8	1,659	5.3	286
Agder	5.7	240,293	8.8	1,148	5.4	291
Vestland	11.8	496,680	7.3	952	21.8	1,181
Trøndelag	8.9	372,109	6.5	843	4.6	247
Troms and Finnmark	4.6	193,656	1.5	196	4.5	242
Unknown	0.02	802	0.03	4	0.0	0
**Risk for severe COVID-19** ^a^						
High	2.7	114,937	1.0	131	0.7	40
Medium	18.8	790,552	9.9	1,290	8.0	433
Low	78.5	3,299,400	89.1	11,580	91.3	4,957
**Type of vaccine among partly and fully vaccinated individuals^b^**						
Comirnaty	81.3	2,678,991	67.3	540	79.9	1,731
Spikevax	11.6	383,632	7.2	58	14.0	304
Vaxzevria	0.1	4,425	22.2	178	0.5	11
Vaxzevria + mRNA^c^	4.0	131,323	3.4	27	4.1	89
mRNA mixed^d^	3.0	97,313	0	0	1.5	32

Alpha: Phylogenetic Assignment of Named Global Outbreak (Pango) lineage designation B.1.1.7; COVID-19: coronavirus disease; Delta: B.1.617.2; SARS-CoV-2: severe acute respiratory syndrome coronavirus 2.

^a^ Risk for severe disease based on underlying comorbidities that are associated with a moderate or high risk of serious illness regardless of age (further details provided in the Supplement part 1).

^b^ Among the whole study population (n = 4,204,859), 3,295,684 individuals were considered partly or fully vaccinated on 15 August 2021. The types of vaccines for individuals diagnosed with Alpha and Delta variants include only doses administered before the individual tested positive for SARS-CoV-2 (for those infected with the Alpha variant (n = 803) and with the Delta variant (n = 2,167)). Comirnaty: BioNTech-Pfizer, Mainz, Germany/New York, United States; Vaxzevria: AstraZeneca, Cambridge, United Kingdom; Spikevax: mRNA-1273, Moderna, Cambridge, United States.

^c^ Vaxzevria was discontinued in Norway on 11 March 2021 and those who received their first dose were offered a second dose of either Comirnaty or Spikevax.

^d^ In Norway the combination of vaccine doses of Comirnaty and Spikevax has been administered.

## Study population characteristics and epidemiological situation

**Figure 1:**
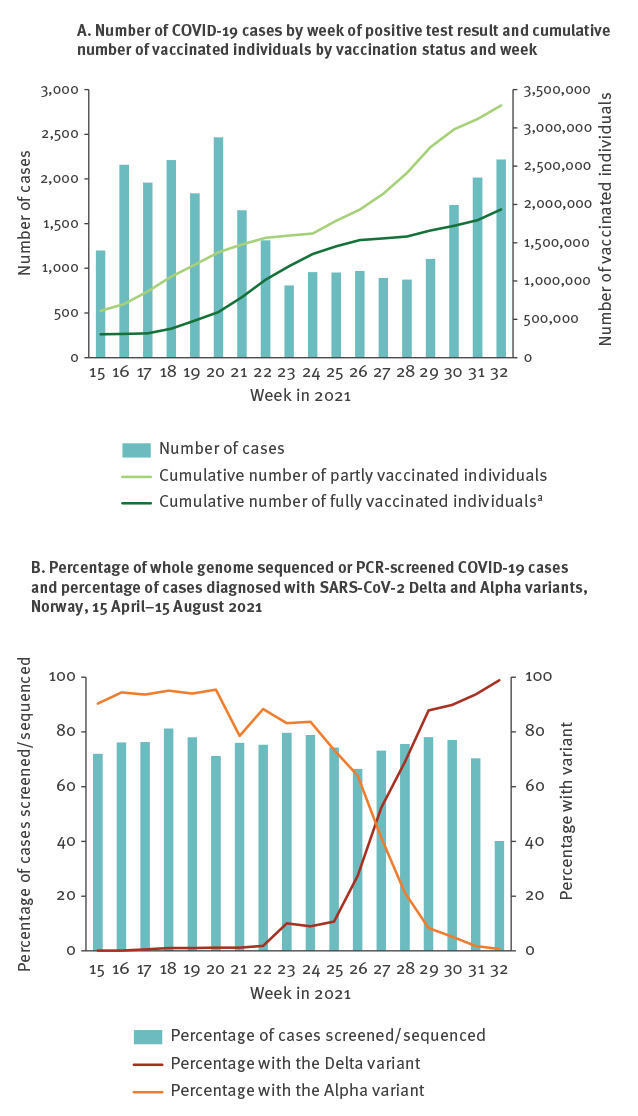
Number of notified cases of COVID-19 by week of positive test result and cumulative number of vaccinated individuals by vaccine status and week; percentage of whole genome sequenced or PCR-screened COVID-19 cases and percentage of cases diagnosed with SARS-CoV-2 Delta and Alpha variants, Norway, 15 April–15 August 2021

COVID-19: coronavirus disease; SARS-CoV-2: severe acute respiratory syndrome coronavirus 2.

In total, we included 4,204,859 individuals of whom 32.4% (1,360,772) were partly vaccinated and 46.0% (1,934,912) were fully vaccinated at the end of follow-up. Characteristics of the study population are shown in Table 1 while the evolution of the epidemic and vaccination coverage is shown in Figure 1 (more information on the vaccination status of the study population can be found in the Supplement, part 2).

Panel A shows number of COVID-19 cases by week of positive test result and cumulative number of vaccinated individuals by vaccination status and week.

Panel B shows percentage of whole genome sequenced or PCR-screened COVID-19 cases and percentage of cases diagnosed with SARS-CoV-2 Delta and Alpha variants, Norway, 15 April–15 August 2021.

^a^ Included in the number of partly vaccinated individuals.

As our study starts from 15 April the actual numbers for week 15 are higher than shown here.

By 15 August 2021, 27,284 individuals had tested positive for SARS-CoV-2. The Delta variant was identified in 5,430 (0.13%) individuals, of whom 1,609 (29.6%) were partly vaccinated and 558 (10.3%) were fully vaccinated at the time of sampling. The Alpha variant was identified in 13,001 (0.31%) individuals, of whom 596 (4.6%) were partly vaccinated and 207 (1.6%) fully vaccinated. The representativeness of cases with data on variants compared with all reported cases of SARS-CoV-2 infection is shown in the Supplement, part 3.

### Vaccine effectiveness against the SARS-CoV-2 Delta and Alpha variants

Using vaccination status as a time-dependent covariate, and explicit time to account for changes in the baseline hazard over time in a Cox proportional hazards model, we estimated the VE against infection with the Delta and Alpha variants, adjusting for factors which were associated with the likelihood of being vaccinated and being infected with the Alpha or Delta variant, namely age, sex, country of birth, county of residence and underlying comorbidities associated with increased risk of severe COVID-19 (Table 1, Supplement, part 2).

The adjusted VE against infection with the Delta variant was 22.4% (95% CI: 17.0–27.4) among partly vaccinated and 64.6% (95% CI: 60.6–68.2) among fully vaccinated, compared with 54.5% (95% CI: 50.4–58.3) and 84.4% (95% CI: 81.8–86.5), respectively, against the Alpha variant (Table 2). Similarly, a separate analysis stratified by age (Supplement, part 4) showed that the VE against infection with the Delta variant for fully vaccinated individuals was significantly lower than against the Alpha variant, although data within each strata are limited and results should be interpreted with caution. As a sensitivity analysis, we kept all study participants, but excluded all days between the day of the first dose and 21 days after the first dose for each participant. This excluded all follow-up time (time at risk) and infections from the period where individuals had received the vaccine but were not yet considered partly vaccinated and may have had some protection. This approach slightly increased the overall VE estimates (Supplement, part 5).

**Table 2:** Crude and adjusted vaccine effectiveness against infection with the Delta and Alpha variants of SARS-CoV-2, Norway, 15 April−15 August 2021 (n = 18,431)

	Events	Rate^a^	Crude VE		Adjusted VE^b^	
			VE (%)	95% CI	VE (%)	95% CI
**Delta variant** ** **						
Unvaccinated	3,263	10.98	Ref			
Partly vaccinated	1,609	18.85	36.9	32.9–40.7	22.4	17.0–27.4
Fully vaccinated	558	4.09	85.7	84.4–87.0	64.6	60.6–68.2
**Alpha variant** ** **						
Unvaccinated	12,198	41.06	Ref			
Partly vaccinated	596	6.98	74.6	72.4–76.6	54.5	50.4–58.3
Fully vaccinated	207	1.52	93.0	92.0–93.9	84.4	81.8–86.5

Alpha: Phylogenetic Assignment of Named Global Outbreak (Pango) lineage designation B.1.1.7; CI: confidence interval; Delta: B.1.617.2; SARS-CoV-2: severe acute respiratory syndrome coronavirus 2; VE: vaccine effectiveness.

^a^ Incidence rate per 1,000,000 person-days.

^b^ Adjusted for age, sex, county of residence, country of birth and underlying comorbidities increasing the risk for severe COVID-19.

Cox proportional hazard models were implemented using explicit time accounting for changes in the baseline hazard over time.

By 15 August 2021, 84 (1.6%) of 5,430 individuals infected with the Delta variant were hospitalised with COVID-19 as the main diagnosis. Of these, 62 were unvaccinated, 13 were partly vaccinated and nine were fully vaccinated, comprising 1.9% of unvaccinated, 0.8% of partly vaccinated and 5.3% of fully vaccinated individuals infected with the Delta variant (Table 2). Among the 13,001 infected with the Alpha variant, 382 (2.9%) individuals were hospitalised of whom 353 were unvaccinated, 14 were partly vaccinated and 10 were fully vaccinated, comprising 2.9% of unvaccinated, 2.5% of partly vaccinated and 5.3% of fully vaccinated individuals infected with the Alpha variant (Table 2). The median age of fully vaccinated cases that were hospitalised among those infected with the Delta variant was 87 (interquartile range (IQR): 58–89) years and six of these had underlying comorbidities, while the median age of those with the Alpha variant was 77 (IQR: 56–82) years and nine had underlying comorbidities.

Up to 15 August 2021, five individuals with the Delta variant died with COVID-19, of whom one was unvaccinated, one was partly vaccinated and three were fully vaccinated. The median age of all cases who died with the Delta variant was 75 (IQR: 75–93) years, and two had underlying comorbidities. In the same period, 25 individuals infected with the Alpha variant died with COVID-19, of whom 12 were unvaccinated, five were partly vaccinated and eight were fully vaccinated. The median age for the fully vaccinated individuals who died with the Alpha variant was 85 (IQR: 82–94) years, and six had underlying comorbidities. Vaccine effectiveness against severe disease was not calculated because of the low number of hospitalisations and deaths.

### Ethical statement

Ethical approval was granted by Regional Committees for Medical and Health Research Ethics (REC) South East (reference number 122745).

## Discussion

The VE against infection was lower for the Delta variant compared with the Alpha variant among both partly and fully vaccinated individuals. Nevertheless, fully vaccinated individuals had lower risk of infection with the Delta (HR 0.35 (95% CI: 0.32–0.39)) and Alpha variant (HR 0.16 (0.13–0.18)) compared to unvaccinated individuals. This is in line with previous studies which have assessed vaccine effectiveness against symptomatic infections [4,5,11]. Our estimates of VE should, however, be interpreted with caution since they are based on observational data. In addition, comparisons with other studies should take into account different study designs, e.g. in our study we included both symptomatic and asymptomatic infections. Another point to keep in mind is that in Norway the main vaccines administered were the mRNA vaccines Comirnaty and Spikevax.

COVID-19 testing in Norway is not limited to symptomatic patients. It is available and free of charge for everyone including those with mild symptoms, close contacts and individuals in quarantine. Testing activity for SARS-CoV-2 is high and since January 2021, most reported cases have either been screened with a PCR variant assay or sequenced [9,12]. Approximately 55% of reported cases have been screened successfully since January and 71% since April [6]. The testing strategy in Norway was enhanced from mid-February 2021, with increased contact tracing and more extensive testing during the period when the Alpha and Beta variants were more prevalent [9]. This testing strategy has facilitated the equal detection of all variants of SARS-CoV-2 and assisted in limiting bias in the comparison of VE against Alpha and Delta variants in our study.

Norway started COVID-19 vaccination on 27 December 2020, focusing on individuals over 65 years, health care workers, elderly people and individuals with increased risk of severe COVID-19 due to underlying comorbidities [13] (Supplement, part 1). From March 2021, parts of Oslo and other municipalities mainly in Viken county have been prioritised due to increased disease burden. By 15 August, 87% of the adult population aged 18 years or older had received one dose and 52% had received two doses, primarily of mRNA-vaccines [6]. The prioritisation of some groups has resulted in varying trends of vaccination uptake across counties, age and risk groups. While we have adjusted for this by including them as covariates in the Cox model, the impact of this imbalance could potentially bias the results. Furthermore, there have recently been reports of declining VE against infection over time [14]. Although our methods should adjust for changes in underlying risks over time, such a decline may confound our analysis and bias the VE against Delta downwards, as most infections with the Delta variant occurred towards the end of follow-up in our study.

The substantial difference in VE between partly and fully vaccinated individuals emphasises the importance of ensuring high coverage for the second dose to reduce transmission. At the same time, although protection against infection with the Delta variant is suboptimal after the first dose, other countries have reported high VE against severe outcomes (above 78% for the different types of vaccines) and reduced risk of severe disease with the Delta variant even after the first dose [5,11]. The small number of hospital admissions and deaths related to the Delta variant among fully and partly vaccinated individuals in Norway support these reports of high VE against severe disease.

The proportions of individuals with severe outcomes (hospitalisation and/or death) in our study cannot be directly compared between variants because most Delta cases were diagnosed at the end of follow-up, and some of these cases may have developed severe disease after the end of follow-up. Additionally, the probability of vaccinated persons developing severe disease is confounded by the fact that a larger proportion of elderly people and people with underlying comorbidities are fully vaccinated, were vaccinated earliest and with the shortest interval between first and second dose.

## Conclusion

Data on protection against infection and severe disease are crucial to guide future vaccination strategy. Effectiveness of COVID-19 vaccines against SARS-CoV-2 infection with both the Delta and Alpha variants appears to be considerable among fully vaccinated people in Norway. Suboptimal protection against infection after the first dose supports efforts to ensure high uptake for the second dose.

## Supplementary Data

896000 Supplement
