# Supplementary material for: Vaccine effectiveness against infection with the Delta (B.1.617.2) variant, Norway, April to August 2021
Source: Euro Surveill. 2021 Sep 2;26(35):2100793. doi: 10.2807/1560-7917.ES.2021.26.35.2100793 (PMC8414959; doi:10.2807/1560-7917.ES.2021.26.35.2100793)
Supplement: Supplement [file 21-00793_SEPPALA_Supplement.pdf]

Supplementary materials: Vaccine effectiveness against infection with the Delta (B.1.617.2) variant, Norway, April to August 2021

This supplementary material is hosted by Eurosurveillance as supporting information alongside the article "Vaccine effectiveness against the Delta (B.1.617.2) variant, Norway, April to August 2021, on behalf of the authors, who remain responsible for the accuracy and appropriateness of the content. The same standards for ethics, copyright, attributions and permissions as for the article apply. Supplements are not edited by Eurosurveillance and the journal is not responsible for the maintenance of any links or email addresses provided therein.

Part 1. Data sources and definition of medium and high risk for severe course of COVID-19

The national identity number was essential to link data from all registries used in the analysis (Table S1).

Table S1. Data sources in the Norwegian preparedness registry BeredtC19 used in this study and individualized data retrieved from each source.

Data source	Data content
Norwegian Surveillance System for Communicable Diseases (MSIS)	Confirmed cases of COVID-19, COVID-19 associated deaths
MSIS laboratory database	Results of PCR screening assays or whole genome sequencing of samples positive for SARS-CoV-2
The Norwegian Intensive Care and Pandemic Registry	Hospitalisations with confirmed COVID-19 as main cause of hospitalisation
The Norwegian Immunisation Registry (SYSVAK)	COVID-19 vaccine doses given
The National Population Register	Registration status (alive/dead)
Norwegian Patient Registry (NPR) and Norway Control and Payment of Health Reimbursement database (KUHR)	ICD-10 and ICPC-2 codes on underlying comorbidities

Some people have underlying comorbidities that cause them to have a moderate or high risk of severe COVID-19 regardless of age. These individuals have been prioritized early for vaccination.

The underlying comorbidities that have been defined as increasing the risk of severe COVID-19 are divided into two groups:

<u>Risk group 1 (high risk)</u> includes people with diseases/conditions that carry a high risk of severe COVID-19, also in younger individuals. These comorbidities include having received an organ transplant, immunodeficiency, hematological cancer in the last five years, other active cancers, ongoing or recently discontinued treatment for cancer (especially immunosuppressive therapy, radiation therapy to the lungs or cytotoxic drugs), neurological or neuromuscular diseases that cause impaired cough or lung function (e.g., ALS and cerebral palsy), Down syndrome and chronic kidney disease, or significant renal impairment.

<u>Risk group 2 (medium risk)</u> includes people with diseases/conditions that entail a moderate risk of severe COVID-19. This includes chronic liver disease or significant hepatic impairment, immunosuppressive therapy as in autoimmune diseases, diabetes, chronic lung disease including cystic fibrosis and severe asthma which have required the use of high dose inhaled or oral steroids within the past year, obesity with a body mass index (BMI) of ≥35 kg/m2, dementia, chronic heart and vascular disease (with the exception of high blood pressure) and stroke.

Part 2. Vaccination status at the end of study

In this rapid communication, we have defined vaccination status as:

- Unvaccinated: unvaccinated, <21 days after 1st vaccine dose
- Partly vaccinated: ≥21 days after 1st vaccine dose, <7 days after 2nd vaccine dose
- Fully vaccinated: ≥7 days after 2nd vaccine dose

Table S2 shows the vaccination status for different subgroups of the study population. Here, we defined vaccination status at the end of the study period – i.e. it does not take into account the vaccination status at the time of infection of individuals who tested positive for SARS-CoV-2 during the study period.

Table S2. Vaccination status by sex, age group, country of birth, county of residence and risk for severe COVID-19, and types of vaccines administered, Norway, 15 April – 15 August 2021.

Vaccination status at 15 Aug 2021					
	**Study population** (4,204,859)	**Partly vaccinated** (1,360,772)		**Fully vaccinated** (1,934,912)	
**Characteristics**	n	%	n	%	n
**Sex**					
Female	2,096,298	30.4	636,727	50.8	1,064,432
Male	2,108,561	34.3	724,045	41.3	870,480
**Age**					
18-24	448,515	51.4	230,406	17.8	79,779
25-34	729,432	37.0	269,609	16.9	123,471
35-44	689,615	44.7	308,062	23.2	159,687
45-54	729,622	46.4	338,472	39.6	288,925
55-64	642,009	30.5	195,771	60.7	389,668
65-74	534,921	1.9	9,963	92.6	495,390
75-84	313,779	1.8	5,575	93.4	292,969
≥85	116,936	2.5	2,914	89.8	105,023
**Country of birth**					
Norway	3,137,823	34.6	1,085,811	46.7	1,466,657
Outside of Norway	1,066,410	25.8	274,801	43.9	468,010
Unknown	626	25.6	160	39.1	245
**County of residence**					
Oslo	538,714	38.6	208,183	45.3	244,123
Rogaland	366,725	33.5	122,823	41.2	151,037
Møre and Romsdal	208,663	26.3	54,885	45.9	95,673
Nordland	192,682	24.4	46,929	46.4	89,369
Viken	960,836	38.1	366,347	46.4	446,234
Innlandet	298,949	25.9	77,376	47.7	142,646
Vestfold and Telemark	334,750	33.6	112,516	46.6	155,914
Agder	240,293	25.6	61,474	50.4	121,074
Vestland	496,680	28.6	141,807	48.4	240,596
Trøndelag	372,109	32.1	119,268	43.6	162,152
Troms and Finnmark	193,656	25.3	48,967	44.3	85,853
Unknown	802	24.6	197	30.1	241
**Risk for severe COVID-19**[a]					
High	114,937	5.9	6,795	87.7	100,828
Medium	790,522	10.6	83,625	80.5	636,590
None	3,299,400	38.5	1,270,352	36.3	1,197,494

Type of vaccine[b]					
Comirnaty [c]	2,974,727	81.1	1,104,046	81.4	1,574,945
Spikevax	441,038	14.4	195,348	9.7	188,284
Vaxzevria	4,450	0.3	3,347	0.1	1,078
Vaxzevria+ mRNA [d]	131,324	0.2	252	6.8	131,071
mRNA mixed [e]	97,400	4.3	57,779	2.0	39,534

[a] *Risk for severe disease based on underlying comorbidities that are associated with a moderate or high risk of serious illness regardless of age (further details provided in this supplement, part 1).*

[b] *For types of vaccines, percentages have been calculated per column, and not per row as for the other characteristics presented in the table.*

[c] *Comirnaty: BioNTech-Pfizer, Mainz, Germany/New York, United States; Vaxzevria: AstraZeneca, Cambridge, United Kingdom; Spikevax: mRNA-1273, Moderna, Cambridge, United States.*

[d] *Vaxzevria was discontinued in Norway on 11 March 2021 and those who received their first dose were offered a second dose of either Comirnaty or Spikevax.*

[e] *In Norway the combination of vaccine doses of Comirnaty and Spikevax has been administered.*

Part 3. Assessment of representativeness of screened cases with available variant data

We assessed the representativeness of the screened cases by comparing the characteristics of the screened cases and notified cases among our study population.

We found differences between cases who were screened for virus variants with regards to county of residence, sampling week, and hospitalisation (Table S3). Differences in county and sampling week reflect the evolution of the outbreak as well as the introduction of PCR screening methodology for virus variants at the primary diagnostic laboratories. Differences in the sampling week are also influenced by the latest weeks not being fully updated regarding the information on detected variants since there is a delay in analysing the samples (and expected to be more updated in the coming weeks). The proportion of cases screened among hospitalised cases was slightly higher than among those not hospitalised (77% vs 72%) and slightly higher among non-Norwegian born (74 vs 72 %). The above differences were considered minor for our study aim.

Table S3. Characteristics of reported and screened cases of SARS-CoV-2, Norway, 15 April to 15 August 2021.

		All reported cases		Screened cases		
**Characteristics**		**N**	**%**	**N**	**%**	**% of all reported**
**Total**		27,284	100	19,721	100	72 %
**Sex**	**Female**	12,560	46	9,146	46	73 %
	**Male**	14,724	54	10,575	54	72 %
						P-value=0.067
**Age group**	**18-24**	8,513	31	6,175	31	73 %
	**25-34**	6,795	25	4,921	25	72 %
	**35-44**	4,968	18	3,567	18	72 %
	**45-54**	4,237	15	3,073	16	73 %
	**55-64**	1,851	6.8	1,311	6.7	71 %
	**65-74**	578	2.1	439	2.2	76 %
	**75-84**	212	0.8	145	0.7	68 %
	**>=85**	130	0.5	90	0.5	69 %
						P-value= 0.246
**Norwegian born**	**Yes**	17,218	63	12,489	63	73 %
	**No**	10.057	37	7,223	37	72 %

	**Unknown**	9		9		
						P-value = 0.204
**Period of diagnosis**	**Weeks 15-16***	3,357	12 %	2,503	13	75 %
	**Weeks 17-18**	4,169	15	3,279	17	79 %
	**Weeks 19-20**	4,305	16	3,190	16	74 %
	**Weeks 21-22**	2,960	11	2,241	11	76 %
	**Weeks 23-24**	1,763	6.5	1,395	7.1	79 %
	**Weeks 25-26**	1,923	7.1	1,349	6.8	70 %
	**Weeks 27-28**	1,763	6.5	1.307	6.6	74 %
	**Weeks 29-30**	2,812	10	2,166	11	77 %
	**Weeks 31-32**	4,232	16	2,291	12	54 %
						P-value<0.001
**County of residence**	**Oslo**	5,728	21	4,486	23	78 %
	**Rogaland**	2,178	7.9	1,588	8.1	73 %
	**Møre and Romsdal**	815	3.0	403	2.0	49 %
	**Nordland**	475	1.7	202	1.0	43 %
	**Viken**	6,334	23	4,321	22	68 %
	**Innlandet**	1,495	5.5	1,044	5.3	70 %
	**Vestfold and Telemark**	2,602	9,5	1,959	9.9	75 %
	**Agder**	2,190	8.0	2.018	10	92 %
	**Vestland**	2,877	11	2,148	11	75 %
	**Trøndelag**	1,480	5.4	1,104	5.6	75 %
	**Troms and Finnmark**	1,104	4.1	444	2.3	40 %
	**Unknown**	6	0.02	4	0.02	67 %
						P-value<0.001
**Hospitalised**	**Yes**	640	98	491	98	72 %
	**No**	26,644	2.4	19,230	2.5	77 %
						P-value=0.011

*Note: the p-values presented are from chi-square tests (the variable categories "unknown" were excluded in these). *Here we have restricted the period after 15th of April (since mid of week 15) and therefore the actual number of cases reported and screened during week 15 is higher than reported here.*

Part 4. Vaccine effectiveness estimates for age groups 18-44, 45-64 and 65+ years

Here we present vaccine effectiveness estimates against infection with the Delta and Alpha variants separately for the age groups 18-44, 45-64 and 65+ years. As for the analyses presented in the main text, we used vaccination status as a time-dependent covariate, and explicit time to account for changes in the baseline hazard over time in a Cox proportional hazards model, adjusting for sex, county of birth, county of residence, and underlying comorbidities associated with increased risk of severe COVID-19. The results should be interpreted with caution due to data paucity.

Table S4. Crude and adjusted vaccine effectiveness (VE) against infection with the Delta and Alpha variants of SARS-CoV-2 for each age group, Norway, 15 April - 15 August 2021 (n = 18,431).

					Crude VE		Adjusted VE[b]	
**Variant**	**Age group**	**Vaccination status**	**Events**	**Rate[a]**	**VE (%)**	**95% CI**	**VE (%)**	**95% CI**
**Delta**								
	18-44	Unvaccinated	2924	16.02			*Ref.*	
		Partly vaccinated	1147	41.95	14.4	8.0 – 20.3	24.3	18.3 – 29.7

		Fully vaccinated	240	11.78	67.9	63.4 – 71.9	67.0	62.1 – 71.1
	45-64	Unvaccinated	305	3.29			*Ref.*	
		Partly vaccinated	448	12.14	21.9	8.5 – 33.3	10.5	-4.9 – 23.7
		Fully vaccinated	175	5.36	65.5	58.1 – 71.6	64.0	55.8 – 70.6
	65+	Unvaccinated	31	1.62			*Ref.*	
		Partly vaccinated	13	0.67	16.9	-62.4 – 57.5	12.9	-70.7 – 55.6
		Fully vaccinated	137	1.70	70.9	56.8 – 80.3	70.0	55.4 – 79.8
**Alpha**								
	18-44	Unvaccinated	9140	50.07			*Ref.*	
		Partly vaccinated	249	9.10	57.8	52.0 – 62.8	56.5	50.6 – 61.7
		Fully vaccinated	54	2.65	89.3	86.1 – 91.8	88.9	85.5 – 91.5
	45-64	Unvaccinated	2805	30.29			*Ref.*	
		Partly vaccinated	213	5.77	52.6	45.3 – 58.9	56.8	50.0 – 62.6
		Fully vaccinated	46	1.41	84.0	78.4 – 88.1	85.4	80.4 – 89.2
	65+	Unvaccinated	219	11.42			*Ref.*	
		Partly vaccinated	124	6.40	40.4	24.4 – 53.0	44.5	29.7 – 56.2
		Fully vaccinated	107	1.33	68.7	59.0 – 76.0	72.9	64.5 – 79.3

Alpha: Phylogenetic Assignment of Named Global Outbreak (Pango) lineage designation B.1.1.7; CI: confidence interval; Delta: B.1.617.2; SARS-CoV-2: severe acute respiratory syndrome coronavirus 2; VE: vaccine effectiveness.

[a] Incidence rate per 1,000,000 person-days

[b] Adjusted for sex, county of residence, country of birth and underlying comorbidities increasing the risk for severe COVID-19. Cox proportional hazard models were implemented using explicit time accounting for changes in the baseline hazard over time.

Part 5. Sensitivity analysis of vaccine effectiveness estimates

In this rapid communication, we have defined vaccination status as:

- Unvaccinated: unvaccinated, <21 days after 1st vaccine dose
- Partly vaccinated: ≥21 days after 1st vaccine dose, <7 days after 2nd vaccine dose
- Fully vaccinated: ≥7 days after 2nd vaccine dose

These definitions, while widely used in analyses of VE, might bias the VE estimate downwards if the vaccines induce some protection prior to day 21 after first dose. As a sensitivity analysis, we looked at whether this potential bias could change our results and conclusion. In this analysis, we kept all study participants, but excluded all follow-up time between the day of the first dose and 21 days after the first dose. This excluded 925 Delta infection events and 910 Alpha infection events. This approach slightly increased the VE estimates, and the resulting crude and adjusted VE estimates are presented in Table S5.

Table S5. Crude and adjusted vaccine effectiveness (VE) against infection with the Delta and Alpha variants of SARS-CoV-2, Norway, 15 April - 15 August 2021 (n = 16,596).

				**Crude VE**		**Adjusted VE[b]**	
**Variant**	**Vaccination status**	**Events**	**Rate[a]**	**VE (%)**	**95% CI**	**VE (%)**	**95% CI**
**Delta**							
	Unvaccinated	2,338	9.70			*Ref.*	
	Partly vaccinated	1,609	18.85	46.2	42.5 – 49.7	32.2	27.1 – 37.0
	Fully vaccinated	558	4.09	87.7	86.5 – 88.8	68.8	65.2 – 72.0
**Alpha**							
	Unvaccinated	11,288	46.84			*Ref.*	
	Partly vaccinated	596	6.98	76.1	74.0 – 78.0	56.9	52.9 – 60.6
	Fully vaccinated	207	1.52	93.4	92.4 – 94.2	85.4	82.9 – 87.4

Alpha: Phylogenetic Assignment of Named Global Outbreak (Pango) lineage designation B.1.1.7; CI: confidence interval; Delta: B.1.617.2; SARS-CoV-2: severe acute respiratory syndrome coronavirus 2; VE: vaccine effectiveness.

[a] *Incidence rate per 1,000,000 person-days*

[b] *Adjusted for age, sex, county of residence, country of birth and underlying comorbidities increasing the risk for severe COVID-19.*

Cox proportional hazard models were implemented using explicit time accounting for changes in the baseline hazard over time
